# The Occurrence and Bioactivities of *Amaryllidaceae* Alkaloids from Plants: A Taxonomy-Guided Genera-Wide Review

**DOI:** 10.3390/plants14131935

**Published:** 2025-06-24

**Authors:** G. David Lin, Pinky Vishwakarma, Paul N. Smith, Rachel W. Li

**Affiliations:** 1 Research School of Chemistry, The Australian National University, Acton 2601, Australia; pinky.vishwakarma@anu.edu.au; 2 School of Medicine and Psychology, The Australian National University, Acton 2601, Australiarachel.li@anu.edu.au (R.W.L.); 3 John Curtin School of Medical Research, The Australian National University, Acton 2601, Australia

**Keywords:** *Amaryllidaceae*, alkaloids, occurrence, galanthamine, anti-Alzheimer’s, anti-acetylcholinesterase, anti-inflammation, bioactivity, drug discovery

## Abstract

The distribution of *Amaryllidaceae* alkaloids, with a focus on their chemodiversity, has been reported previously, but not at a genera-wide diversity level. This review provides a comprehensive survey of the occurrence of *Amaryllidaceae* alkaloids across the genera of the *Amaryllidaceae* family. This survey is taxonomically guided by the National Center for Biotechnology Information (NCBI) Taxonomy Browser, with targeted keyword searches conducted in the Chemical Abstracts Service (CAS) SciFinder-n and PubMed. The family *Amaryllidaceae* comprises over 1214 species across three subfamilies: *Agapanthoideae* (1 genus, 5 species), *Allioideae* (3 genera plus 11 subgenera, 617 species), and *Amaryllidoideae* (58 genera plus 13 subgenera, 592 species). *Amaryllidaceae* alkaloids have been identified exclusively in 36 of the 58 genera and 6 of the 13 subgenera within the *Amaryllidoideae* subfamily. To date, more than 600 *Amaryllidaceae* alkaloids have been isolated, predominantly from this subfamily—hence the designation “*Amaryllidaceae* alkaloids”. These alkaloids display a wide spectrum of biological activities, including acetylcholinesterase inhibition, anti-inflammatory, antioxidant, antimicrobial, antidiabetic, and anticancer effects. A notable example is galanthamine (also known as galantamine), an FDA-approved drug marketed under the brand names Reminyl™ (Janssen Research Foundation, Beerse, Belgium, 2001) and Razadyne™ (Johnson & Johnson Pharmaceutical Research, New Brunswick, NJ, USA, 2004) for the treatment of mild to moderate Alzheimer’s disease, due to its potent acetylcholinesterase-inhibitory activity. Galanthamine has been isolated from species belonging to the genera *Cyrtanthus*, *Galanthus*, *Leucojum*, *Lycoris*, *Narcissus*, *Ungernia*, *Chlidanthus*, *Crinum*, *Eucharis*, *Eustephia*, *Pancratium*, and *Phaedranassa*. Lycorine is another widely distributed alkaloid found across multiple genera, and it has been extensively studied for its diverse bioactivities. Given the remarkable chemical diversity and bioactivity of *Amaryllidaceae* alkaloids, along with the many underexplored genera and species, further research into *Amaryllidaceae* species and their alkaloids is strongly warranted to support the discovery and development of novel therapeutic agents.

## 1. Introduction

Alkaloids can be classified into several categories, including heterocyclic alkaloids; alkaloids with exocyclic nitrogen atoms; aliphatic amines; putrescine-, spermidine-, and spermine-derived alkaloids; peptide alkaloids; terpene and steroid alkaloids; and dimeric (bis-)alkaloids [1]. Among these, *Amaryllidaceae* alkaloids represent one of the most common and pharmacologically significant classes of heterocyclic alkaloids found in the plant kingdom, often regarded as nature’s gift to humanity. To date, over 636 *Amaryllidaceae* alkaloids have been isolated and identified from the *Amaryllidaceae* family [2], hence the name. Notably, galanthamine (or galantamine) is a well-known example that was among the earliest drugs used clinically to treat mild to moderate Alzheimer’s disease, following its FDA approval under the brand names Reminyl™ (Janssen Research Foundation, Beerse, Belgium, 2001) and Razadyne™ (Johnson & Johnson Pharmaceutical Research, New Brunswick, NJ, USA, 2004). A recent systemic review confirms that galantamine (at recommended doses of 16 mg to 24 mg daily) slows a decline in memory and the ability to perform self-care activities at 6 months and 2 years after treatment for people with dementia due to Alzheimer’s disease [3].

Although the distribution of the *Amaryllidaceae* has been reported in an excellent review of chemodiversity, chemotaxonomy, and chemoecology [2], it was focused on the chemodiversity of 17 types of *Amaryllidaceae* alkaloids. This review aims to conduct a genera-wide survey on the occurrence of *Amaryllidaceae* alkaloids in the *Amaryllidaceae* family—covering its genera and species—as listed in the Taxonomy Browser of the National Center for Biotechnology Information (NCBI) (accessed on 31 January 2025). The presence of *Amaryllidaceae* alkaloids was investigated using a combination of keywords, *Amaryllidaceae*, alkaloids, occurrence, isolation, and detection, alongside the names of the family, genera, subgenera, and species. Searches were conducted using the Chemical Abstracts Service (CAS) SciFinder-n database, up to 31 January 2025. In addition, this review discusses reported biological and pharmacological activities based on literature searches performed via PubMed (also up to 31 January 2025) using a combination of *Amaryllidaceae* and/or alkaloid keywords with bioactivity-related keywords including anti-acetylcholinesterase, anti-inflammatory, antioxidant, antimicrobial, antidiabetic, and anticancer.

## 2. Genera and Species from the Family *Amaryllidaceae*

The *Amaryllidaceae* family, which belongs to the *Asparagales* order, comprises more than 100 genera and approximately 1200 species [4]. These species are widely distributed across tropical and subtropical regions of the world, including Africa, Australia, Eurasia, and the Americas.

The family *Amaryllidaceae* are monocotyledons, perennial herbs with bulbs. Occasionally, in genera *Clivia* and *Cryptostephanus* and some *Scadoxus* species, rhizomes instead of bulbs are observed [5]. According to the NCBI Taxonomy ID 4668 for *Amaryllidaceae* [6], the *Amaryllidaceae* family comprises 1214 species distributed across 62 genera plus 24 subgenera, classified within three subfamilies: *Agapanthoideae*, *Allioideae*, and *Amaryllidoideae* (Table 1).

The 62 genera plus 24 subgenera identified in this review are fewer than the approximately 100 genera estimated by Ji and Meerow [4]. According to the Angiosperm Phylogeny Website, the *Amaryllidaceae* family under the order *Asparagales* comprises 73 genera and 1605 species [7]. It is important to note that the 1214 species listed in Table 1 do not include many unclassified species, which may explain why this number is lower than the 1605 species reported by the Angiosperm Phylogeny Website, but is close to—or slightly higher than—the 1200 species documented by Ji and Meerow. Such discrepancies among different taxonomic classification systems are common and expected.

The NCBI Taxonomy Database includes a disclaimer stating that it is not an authoritative source for nomenclature or classification and advises consulting the relevant scientific literature for the most reliable information. Nevertheless, the database serves as a valuable resource for researchers beyond the field of taxonomy. Accordingly, its classification system for the *Amaryllidaceae* family is utilized in this review as a framework to survey the occurrence of *Amaryllidaceae* alkaloids.

The family *Amaryllidaceae* is divided into three subfamilies: *Agapanthoideae*, *Allioideae*, and *Amaryllidoideae*. The occurrence of alkaloids across these subfamilies and under their genera is the focus of this taxonomic-guided genera-wide review.

**Figure 1:**
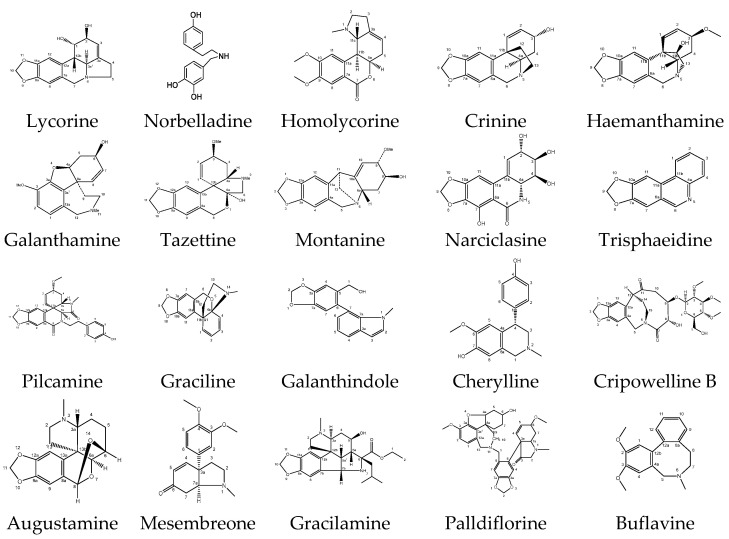
Structures of 20 *Amaryllidaceae* alkaloids representing the diversity of alkaloid types.

Plants in the family *Amaryllidaceae* have yielded more than 636 reported alkaloids, including both structurally characterized and tentatively identified compounds [2,8]. Several attempts have been made to classify the massive number of *Amaryllidaceae* alkaloids. Norbelladine, lycorine, homolycorine, crinine, haemanthamine, narciclasine, tazettine, montanine, and galanthamine form nine major types with a unifying numbering system of the different skeletons [9]. Fifteen types of structures [10] and eighteen types [11] were also grouped. Twenty *Amaryllidaceae* alkaloid types were proposed [12], and they represent the diversity of alkaloid structures, as depicted in Figure 1.

## 3. Alkaloids from Subfamily *Agapanthoideae*

The subfamily *Agapanthoideae* is the first of the three subfamilies within the *Amaryllidaceae* family and includes a single genus, *Agapanthus*, comprising five species, *A. africanus*, *A. campanulatus*, *A. caulescens*, *A. coddii*, and *A. praecox*, as summarized in Table 2.

To date, no specific *Amaryllidaceae* alkaloids have been reported in these five *Agapanthus* species (Table 2). However, one study examining the dichloromethane extract of *Agapanthus campanulatus* roots detected the presence of alkaloids using TLC separation and the Dragondorff reagent [13].

## 4. Alkaloids from Subfamily *Allioideae*

The subfamily *Allioideae* is the second of the three subfamilies within the *Amaryllidaceae* family and includes three genera, *Allieae*, *Gilliesieae*, and *Tulbaghieae*, comprising 617 species (Table 3).

The genus *Allieae* comprises 524 species, none of which have been found to contain *Amaryllidaceae* alkaloids, based on the SciFinder search described above. However, three studies have reported the presence of other types of alkaloids, including alline [16], 1,2,3,4-tetrahydro-4-hydroxy-4-quinolin carboxylic acid [15], and two indole-type alkaloids—canthin-6-one and 8-hydroxy-canthin-6-one [14].

The genus *Gilliesieae* includes nine subgenera—*Beauverdia*, *Gilliesia*, *Latace*, *Leucocoryne*, *Miersia*, *Nothoscordum*, *Speea*, *Tristagma*, and *Zoellnerallium*—with a total of 75 species. To date, no *Amaryllidaceae* alkaloids have been reported in any of these species.

The genus *Tulbaghieae* consists of two subgenera: *Prototulbaghia* and *Tulbaghia* (Table 2). The subgenus *Prototulbaghia* includes a single species, *P. siebertii*, with no reports of *Amaryllidaceae* alkaloids. The subgenus *Tulbaghia* comprises 17 species, among which two species, *T. cernua* and *T. ludwigiana*, were reported to exhibit acetylcholinesterase (AChE) inhibitory activity at a concentration of 1 mg/mL of ethanol extract [17]. However, the active constituents were not identified or isolated.

## 5. Alkaloids from Subfamily *Amaryllidoideae*

The subfamily *Amaryllidoideae* is the third of the three subfamilies within the *Amaryllidaceae* family and includes 58 genera plus 13 subgenera with a total of 592 species (Table 4). The thirteen subgenera include three—*Clinanthus*, *Pamianthe*, and *Paramongaia*—which belong to the genus *Clinantheae* and ten—*Famatina*, *Hippeastrum*, *Paposoa*, *Phycella*, *Placea*, *Rhodolirium*, *Rhodophiala*, *Sprekelia*, *Traubia*, and *Zephyranthes*—which fall under the genus *Hippeastreae*.

The subfamily *Amaryllidoideae* is the primary source of *Amaryllidaceae* alkaloids. Due to the widespread occurrence of these alkaloids within this subfamily, each genus and subgenus has been surveyed for the presence of *Amaryllidaceae* alkaloids as reported in the scientific literature (Table 4). The alkaloids from each genus or subgenus within the subfamily *Amaryllidoideae* are surveyed and categorized into three groups:

- Isolated *Amaryllidaceae* alkaloids;
- Detected and identified (but not isolated) *Amaryllidaceae* alkaloids;
- No reported *Amaryllidaceae* alkaloids from the subfamily *Amaryllidoideae.*

### 5.1. Isolated Amaryllidaceae Alkaloids from the Subfamily Amaryllidoideae

#### 5.1.1. Genus *Amaryllis*

The genus *Amaryllis* has three species (Table 4), including *A. munita*, *A. paradisicola*, and *A. belladonna*. *Amaryllis belladonna* has been extensively studied for the *Amaryllidaceae* alkaloids. In 1984, a new alkaloid, anhydrolycorinium chloride, was isolated together with the known acetylcaranine, ambelline, and undulatine from *A. bellodonna*. The identification of these new and known alkaloids was characterised using UV-VIS, FT-IR, MS, and proton and carbon NMR. The ED_50_ value against the murine P-388 lymphocytic leukemia was also studied and found to be 1.4, 0.23, and 1.6 µg/mL for anhydrolycorinium chloride, acetylcaranine, and ambelline, respectively [18]. Using HPLC-UV-MS, in 1996, Queckenberg et al. identified nine additional alkaloids which were not reported in *A. belladonna* before, including anhydrolycorin-7-one, 6-OH-buphanisine, 6-OH-crinine, crinine, galanthine, hippadine, ismine, pratorimine, and pratosine [19].

The new alkaloid (−)-amarbellisine was isolated together with five known alkaloids, (−)-lycorine, (−)-pancracine, (+)-vittatine, (+)-11-hydroxyvittatine, and (+)-hippeastrine, from *A. belladonna* L. [20]. Cho and coworkers isolated 1,4-dihydroxy-3-methoxypowellan, distichamine, 11-*O*-acetylambelline, ambelline, acetylcaranine, and hippadine from *Amaryllis belladonna* Steud. bulbs [21]. In one study, four alkaloids, belladine, undulatine, buphanidrine, and acetylcaranine, were isolated from *A. belladonna* [17] and studied for their inhibition of acetylcholine esterase in a search to find a better inhibitor than galanthamine, a clinically useful drug for Alzheimer’s disease. Acetylcaranine was found to be the most active towards acetylcholine esterase (AChE), with an IC_50_ of 11.7 ± 0.7 μM, which is comparable to that of galanthamine (IC_50_ = 6.19 ± 2.60 μM).

#### 5.1.2. Genus *Ammocharis*

The genus *Ammocharis* has six species (Table 4), including *A. angolensis*, *A. baumii*, *A. coranica*, *A. longifolia*, *A. nerinoides*, and *A. tinneana*. From fresh bulbs of *A. tinneana*, sixteen alkaloids were isolated, and 2D NMR techniques were used for the structural elucidation [22]. 6α-Hydroxycrinamidine and 6α-hydroxyundulatine were reported for the first time together with known alkaloids: seven alkaloids have a 1,2-β-epoxide group and are 1,2-β-epoxyambelline, 11-*O*-acetyl-1,2β-epoxyambelline, 6α-hydroxycrinamidine, 6α-hydroxyundulatine, flexinine, 1,2-β-epoxyambelline, and 11-*O*-acetyl-1,2-β-epoxyambelline; six alkaloids are crinine-type, including ambelline, 11-*O*-accetylambelline, xrinine, powelline, buphanidine, and buphanisine, and three lycorine-type alkaloids including lycorine, sternbergine, and 9-*O*-demethylpluviine.

From the bulbs of *A. coranica*, eight alkaloids were isolated: lycorine, acetylcaranine, and crinamine have been reported previously, but 1-O-acetyllycorine, hippadine, 6a-hydroxypowelline, and hamayne were reported from other members of the *Amaryllidaceae* only, and 1-O-acetyl-9-O-demethylpluviine has not been reported previously [23]. In a recent study of *A. coranica*, three new alkaloids, golceptine (lycorine type), 6*α*-hydroxybuphanidrine, and charisine (crinine-type), were isolated, together with the known hippadine, lycorine, caranine, acetylcaranine, and 1-*O*-acetyl-9-*O*-demethylpluviine [24].

#### 5.1.3. Genus *Boophone*

The genus *Boophone* has three species, including *B. disticha*, *B. haemanthoides*, and *B. haemanthoides*. From *B. disticha* crinine, buphanamine, buphanidrine, distichamine, and buphanisine were isolated and characterized [25]. Distichamine, lycorine, undulatine, buphanidrine, ambelline, buphanisine, crinine, and distichaminol were isolated from *B. haemanthoides* [26]. The ethanolic extract of the bulbs of *B. disticha* contains buphanidrine and distichamine, having an antibacterial activity against both Gram-positive and Gram-negative bacteria, with the best MIC of 0.063 mg/mL [27].

*Boophone haemanthoides* was shown to be a novel source of the known β-crinane alkaloids, distichamine, buphanidrine, buphanisine, and crinine. Of note is the presence of distichamine in *B. haemanthoides*, previously identified only in *B. disticha*, and its significance as a distinctive chemotaxonomic marker for the genus *Boophone* [28].

One new compound, 1-*O*-acetylbuphanamine, and four known crinane alkaloids were isolated chromatographically from the ethanol extract of the bulbs of *B. disticha* [29]. From a *B. haemanthoides* extract, three *Amaryllidaceae* alkaloids, distichamine, 1α,3α-diacetylnerbowdine, and hippadine, were isolated [30].

#### 5.1.4. Genus *Brunsvigia*

The genus *Brunsvigia* has eight species, including *B. bosmaniae*, *B. comptonii*, *B. gregaria*, *B. orientalis*, *B. radula*, *B. radulosa*, *B.* cf. *radulosa Spies 7629*, and *B. striata*. From *B. josephin* crinine, buphanisine, buphanidrine, undulatine, 3-*O*-acetylhamayne, hamayne, crinamine, ambelline, and sternbergine were isolated together with a new josephinine [31]. Four known alkaloids, lycorine, 1,2-di-*O*-acetyllycorine, ambelline, and crinine, were isolated from the bulbs of *B. littoralis* [32].

The bulbs of *B. radulosa* yielded a new alkaloid, 1-O-acetylnorpluviine, together with the known alkaloids 1-epideacetylbowdensine, crinamine, crinine, hamayne, lycorine, anhydrolycorin-7-one, and sternbergine [33].

#### 5.1.5. Genus *Calostemma*

The genus *Calostemma* has two species (Table 4), including *C. luteum* and *C. Purpureum*. The bulbs of *C. purpureum* yield a major component of lycorine and three minor components of haemanthamine, crimidine, and powelline [34].

From the *C. purpureum* extract, incartine, lycorine, and galanthine were identified as well as trace amounts of other alkaloids of the lycorine, homolycorine, and tazettine types [35].

#### 5.1.6. Genus *Chlidanthus*

The genus *Chlidanthus* has two species (Table 4), including *C. boliviensis* and *C. fragrans*. In 1956, from 2 kg of *C. fragrans* bulbs, Biot obtained 0.45 g of lycorine, 0.31 g of chlidanthine, and 0.98 g of tazettine [36].

Fifteen known *Amaryllidaceae* alkaloids of five structure types were from the bulbs of *C. fragrans*, using capillary GC-MS. The fifteen alkaloids were identified as galanthamine, chlidanthine, buphanisine, narwedine, belladine, 6-deoxytazettine, *N*-demethylbelladine, 6-*O*-methylpretazzetine, tazzetine, undulatine, lycorine, ambelline, 3-epimacronine, acetylnerbowdine, and bowdesine. The main alkaloids were tazzetine (tazettine-type), chlidanthine (galanthamine-type), belladine (belladine-type), and lycorine (lycorine-type) [37]

Eleven *Amaryllidaceae* alkaloids were isolated from fresh bulbs of *C. fragrans*, and the crinine-type *Amaryllidaceae* alkaloid undulatine showed a promising acetylcholinesterase and prolyl oligopeptidase inhibition activity with IC_50_ values of 23.0 μM and 1.96 mM, respectively [38].

#### 5.1.7. Genus *Clinantheae* (With Three Subgenera)

The genus *Clinantheae* has three subgenera, *Clinathus*, *Pamianthe*, and *Paramongaia* (Table 4). The subgenus *Clinathus* has six species, including *C. coccineus*, *C. humilis*, *C. imasumacc*, *C. incarnatus*, *C. mirabilis*, and *C. ruber*.

The fresh bulb of *Clinanthus microstephium* is extracted with methanol and fractionated using chromatographic techniques. The analysis of the fractions using GC/MS and NMR resulted in the identification of seven known compounds, such as anhydrolycorine, 11,12-dehydroanhydrolycorine, hippeastrine, 1-*O*-butenoyllycorine, 1-*O*-(3-hydroxybutanoyl)lycorine, lycorine, and 6-hydroxymaritidine [39].

The subgenus *Pamianthe* has two species (Table 4), including *P. ecollis* and *P. peruviana*. The subgenus *Paramongaia* has four species (Table 4), including *P. milagroantha*, *P. multiflora*, *P. viridiflora*, and *P. weberbaueri*. No report of *Amaryllidaceae* alkaloids was found from the SciFinder search for both subgenera.

#### 5.1.8. Genus *Clivia*

The genus *Cliva* has nine species (Table 4), including *C. caulescens*, *C. gardenia*, *Clivia* cf. *gardenii* ‘robust’, *C. miniate*, *C. mirabilis*, *C. nobilis*, *C. robusta*, *Clivia* × *cyrtanthiflora*, and *Clivia* × *nimbicola*. Interestingly, *C. caulescens* has rhizomes, instead of bulbs [5].

From *C. caulescens* alkaloids hippeastrine, haemanthamine, (11*S*)-11-hydroxyvittatine, lycorine, and sterbergine were isolated, whereas haemanthamine, haemanthidine in its free base and quaternary salt form, and tazettine in the quaternary salt were isolated from *C. gardenii* [40].

Kornienko and Evidente reported the isolation of clivonine, clivatine, nobilisitine A, and nobilisitine B from *C. miniata* and rystilline, clivimine, nobilisine, and hyamine from *C. nobilis* [41]. Nobilisitine A, nobilisitine B, and 5-O-acetylnobilisitine A were also isolated from *C. nobilis* [42].

Lycorine was isolated as the active component responsible for the pronounced antiviral activity of crude extracts from the roots and leaves of *C. miniata* Regel. In addition, clivimine, clivonine, and cliviamartine were also isolated and characterized; however, these alkaloids did not exhibit antiviral properties [43].

#### 5.1.9. Genus *Crinum*

The genus *Crinum* has 68 species (Table 4), and some important species are highlighted here. The genus has been studied extensively and attracted several comprehensive reviews on the *Crinum* alkaloids, as in *Phytochemistry* [44], the review covering the period from 1985 to 2000 [45] and the review focusing on *C. latifolium* [46].

The Ghosal review presented 46 *Amaryllidaceae* alkaloids isolated from various *Crinum* species [44], whereas the Tram review presented 68 *Amaryllidaceae* alkaloids [45], including ambelline, augustamine, augustine, (−)buphanisine, (−)crinamidine, (+)crinamine, (−)crinine, (−)1,2-di-*O*-acetyllycorine, (−)galanthamine, (+)haemanthamine, hamayne, hippadine, hipeastrine, (−)6-α-hydroxybuphanisine, (+)6-hydroxycrinamine, ismine, (−)lycorine, powelline, pratorimine, pratorinine, pratosine, *O*-methylpratorimine, *O*-methyl-pratorinine, and the 3-epimer of criwelline.

Hippadine, lycorine, hippeastrine, masonine, and crinine have been isolated from *C. firmifolium*; lycorine, crinine, and 6-hydroxycrinamine have been isolated from *C. hardyi*; lycorine, 6-hydroxycrinamine, and pseudolycorine have been isolated from *C. pronkii*; and a novel lycorine-related iminium salt, the 6,7,11b,11c-didehydrolycorinium salt, was isolated from bulbs of both *C. firmifolium* and *C. hardyi* [47].

Novel 4,8-dimethoxy-cripowellin, 4,8-dimethoxy-cripowellin, 9-methoxy-cripowellin, and 4-methoxy-8-hydroxy-cripowellin, along with one known alkaloid, cripowellin, were isolated from the 95% ethanol extract of the bulbs of *C. latifolium*. These compounds exhibited cytotoxic, antimicrobial, antioxidant, and anti-inflammatory activities [48].

Fifteen alkaloids—8α-ethoxyprecriwelline, *N*-desmethyl-8α-ethoxypretazettine, *N*-desmethyl-8β-ethoxypretazettine, bulbispermine, 1-*O*-acetyllycorine, epivittatine, crinamine, 3-*O*-acetylhamayne, 6-hydroxycrinamine, epibuphanisine, powelline, crinine, cherylline, crinamidine, and 1-epideacetylbowdensine—were isolated from *C. bulbispermum* and *C. moorei* [49].

From the bulbs of *C. pratense*, lycorine, 1,2-diacetyllycorine, ambelline, narcissidine, and three phenanthridone alkaloids, viz. hippadine, pratorinine, and anhydrolycorin-7-one, were isolated and characterized [50]. Sixteen additional *Amaryllidaceae* alkaloids were isolated from the bulbs of *C. kirkii* Baker, including two previously unknown compounds, noraugustamine and 4a,*N*-dedihydronoraugustamine [51].

#### 5.1.10. Genus *Crossyne*

The genus *Crossyne* has two species (Table 4), including *C. flava* and *C. guttata*. *Crossyne guttata* yielded the alkaloids crinamine and epibuphanisine [52]. The four compounds isolated from *C. flava* are pancratinine B, bufanidrine, buphanisine, and epibuphanisine [53]

Distichamine was isolated from an ethanolic fraction of *C. guttata* along with two known alkaloids, crinamine and epibuphanisine. Distichamine, a rare β-crinane alkaloid previously reported only from the genus *Boophone*, is now found in the genus *Crossyne*, suggesting that it may be more widespread within the subfamily *Amaryllidaceae* [54].

#### 5.1.11. Genus *Cybistetes*

The genus *Cybistetes* has one species (Table 4), *C. longifolia*, in which three alkaloids, 6-hydroxycrinamine, lycorine, and pseudolycorin were isolated and identified [55].

#### 5.1.12. Genus *Cyrtanthus*

The genus *Crytanthus* has 44 species (Table 4). Two species, *C. elatus* and *C. obliquus*, are showcased with isolated alkaloids here. Zephyranthine and 1,2-O-diacetylzephyranthine were isolated from *C. elatus* [56]. The ethanolic extract of *C. obliquus* (L.f.) Ait yielded a new dinitrogenous alkaloid obliquine, 3*S*,4α*S*,11*S*,10β*S*-3,4,4α,13,11,5,6-heptahydro-5[2-(4-hydroxyphenyl)ethyl]-3-methoxy-13-methyl-[1,3]dioxolo[4,5-g]indolo[3,3α-c]-isoquinolin-12-one, along with five known alkaloids: 11α-hydroxygalanthamine, 3-epimacronine, narcissidine, tazettine, and trisphaeridine [57].

#### 5.1.13. Genus *Eucharis*

The genus *Eucharis* has nine species (Table 4), and *E. amazonica* is showcased with isolated alkaloids here. From 1.2 kg of the dried bulbs and leaves of flowering *E. amazonica*, thirteen alkaloids have been isolated, including 7-methoxyoxoassoanine (12 mg), 6-O-methylpretazettine (6 mg), and apohaemanthamine (49 mg), as newly reported from this species, together with lycorine (64 mg), ismine (26 mg), trisphaeridine (21 mg), tazettine (156 mg), 3-epimacronine (24 mg), haemanthamine (16 mg), galanthamine (81 mg), 3-O-methylgalanthamine (41 mg), vittatine (42 mg), and 8-O-demethylmaritidine (47 mg) [58]. 

#### 5.1.14. Genus *Eustephia*

The genus *Eustephia* has one species (Table 4), *E. yuyuensis*. Three alkaloids were isolated from bulb, with the major alkaloid being lycorine and the minor being galanthamine and galanthine [59].

#### 5.1.15. Genus *Galanthus*

The genus *Galanthus* has 25 species (Table 4). The isolation of galanthamine from various species in the genus *Galanthus* has been reviewed [60]. The phytochemistry of *Amaryllidaceae* alkaloids in *Galanthus* species has been recently reviewed [61].

From *G. nivalis*, Berkov and coworkers isolated and identified two new alkaloids, 3,3′-*O*-(3′,3″-dihydroxybutanoyl)hamayne and 11,3′-*O*-(3′,3″-dihydroxybutanoyl)hamayne, together with six known alkaloids, 3,11-*O*-(3′,3″-dihydroxybutanoyl)hamayne, 3,11,3′-*O*-(3′,3″,3‴-trihydroxybutanoyl)hamayne, 8-*O*-demethylvasconine, tazettine, epimacronine, and ismine. From *G. elwesii*, the group also isolated and identified one new alkaloid, 2-*O*-(3′-hydroxybutanoyl)lycorine, together with two known alkaloids, 2-*O*-(3′-acetoxybutanoyl)lycorine and incartine. In addition, from both species, the known alkaloids hamayne, 11-*O*-(3′-hydroxybutanoyl)hamayne, and lycorine were isolated and identified. All structures were determined using EI-MS, HR-MS, CD, and 1D and 2D NMR, including COSY, NOESY, HMQC, and HMBC [62].

(+)-5-methoxy-9-*O*-demethylhomolycorine, (+)-galwesine, (+)-9-*O*-demethylgalwesine, (+)-16-hydroxygalwesine, (+)-16-hydroxy-9-*O*-demethylgalwesine, and galasine, were isolated from whole plants of *Galanthus elwesii*. Additionally, 12 known alkaloids, (−)-galanthamine, (−)-sanguinine, (−)-leucotamine, (−)-*O*-methylleucotamine, (+)-narwedine, (−)-*N*-demethylgalanthamine, (+)-11-hydroxyvittatine, (+)-9-*O*-demethylhomolycorine, (−)-lycorine, (−)-galanthine, hordenine, and (*E*)-*N*-feruloyltyramine, were also obtained [63].

Tazettine, galanthamine, sanguinine, and haemanthamine were isolated from *G. cilicicus* [64]. Three new alkaloids, oxoincartine, 3,11-*O*-diacetyl-9-*O*-demethylmaritidine, and 11-*O*-acetyl-9-*O*-demethylmaritidine, together with seven known compounds, namely incartine, galanthamine, galanthine, 9-*O*-methylpseudolycorine, *N*,*O*-dimethylnorbelladine, hordenine, and vittatine, were isolated from *G. fosteri* Baker [65].

Two new *Amaryllidaceae* alkaloid *N*-oxides, incartine *N*-oxide and lycorine *N*-oxide, along with one β-carboline alkaloid, 1-acetyl-β-carboline, and six known alkaloids—incartine, *N*-trans-feruloyltyramine, lycorine, *O*-methylnorbelladine, vittatine, and 11-hydroxyvittatine—were isolated from *G. rizehensis* Stern [66]. Also, two new alkaloid *N*-oxides—1-*O*-acetyldihydromethylpseudolycorine *N*-oxide and 11-hydroxyvittatine *N*-oxide—plus ten known alkaloids—arolycoricidine, haemanthamine, *O*-methylnorbelladine, narcidine, dihydrolycorine, 8-*O*-demethylmaritidine, stylopine and protopine, nicotinic acid, and tyramine—were isolated from *G. trojanus* A.P. Davis & N. Ozhatay [67].

#### 5.1.16. Genus *Haemanthus*

The genus *Haemanthus* has 12 species (Table 4) from which *Amaryllidaceae* alkaloids have been isolated. The roots of *H. kalbreyeri* contain a new phenanthridone alkaloid, kalbretorine, and a new glucosyloxy alkaloid, kalbreclasine. Additionally, six known alkaloids, viz. haemanthamine, haemanthidine, hippadine, lycorme, narciclasine, and pratorimine, previously reported from other Amaryllidaceous plants, have now also been isolated from this species [68]. On the other hand, the bulbs of *H. kalbreyeri* contain two new alkaloids, 7-deoxypancratistatine and pancratiside (i.e., pancratistatine-2-*O*-β-D-glucoside), together with known alkaloids, narciclasine, 7-deoxynarciclasine, and pancratistatine [69].

#### 5.1.17. Genus *Hieronymiella*

The genus *Hieronymiella* has two species (Table 4), including *H. argentina* and *H. peruviana*. Nine alkaloids, including galanthamine, sanguinine, and chlidanthine, were isolated from the extract of *H. argentina* [70], and twenty-two alkaloids, most of them of the homolycorine-type and galanthamine-type, were identified in the extracts of *H. peruviana* by means of GC-MS and UPLC-MS/MS [71].

#### 5.1.18. Genus *Hippeastreae* (With 10 Subgenera)

The genus *Hippeastreae* has ten subgenera (Table 4): *Famatina*, *Hippeastrum*, *Paposoa*, *Phycella*, *Placea*, *Rhodolirium*, *Rhodophiala*, *Sprekelia*, *Traubia*, and *Zephyranthes*. Alkaloids have been isolated from four subgenera of *Hippeastrum*, *Rhodolirium*, *Rhodophiala*, and *Zephyranthes*. They are presented below.

#### 5.1.19. *Hippeastrum Species*

From 12 kg of *H. vittatum*, 0.79 g of lycorine, 0.05 g of homolycorine, 0.15 g of chlidathine, 0.95 g of hemanthamine, 0.15 g of hippeastrine, and 0.3 g of vittatine were isolated and identified [36]. Tazettine and haemanthamine were identified from the 0.02% total alkaloids obtained from the bulbs of *H. bifidum* [34].

The application of GC–MS resulted in the identification of two crinine-type alkaloids, aulicine and 3-O-methyl-epimacowine, from the indigenous Brazilian species *H. aulicum* and *H. calyptratum*, respectively. In addition, two alkaloids, 11-oxohaemanthamine and 7-methoxy-O-methyllycorenine, were isolated from *H. aulicum* [72].

A new lycosinine derivative, 9-*O*-demethyllycosinine, was isolated from the endemic Brazilian *Hippeastrum breviflorum* Herb., *Amaryllidaceae*, along with the well-known alkaloids lycosinine and lycorine [73]. *Hippeastrum albiflos* yielded homolycorine, albomaculine, and the *O*-methyl-lycorenium salt. From *H. pauculifolius*, homolycorine, a novel paucamine isolated as a salt, together with the quaternary salts of homolycorine, montanine, and manthidine were obtained. Form *H. deformis*, coccinine, montanine, and the quaternary salt of manthidine were isolated [74].

From the bulbs of the Vietnamese *H. equestre* Herb, two new alkaloids, 3-*O*-emethyltazettine and egonine, have been isolated [75]. Their structures were established by UV, MS, NMR, and CD. It is interesting to note that the identified alkaloid egonine was the alkaloid of the mesembrine-type or mesembrinone-type, usually isolated from *Sceletium* species, not belonging to the family *Amaryllidaceae*. This is why Sceletium alkaloids are sometimes discussed together with *Amaryllidaceae* alkaloids [76].

#### 5.1.20. *Rhodolirium speciosum*

From *R. speciosum*, 1-O-acetyl-5,6-dehydrolycorine, 1-*O*-acetyl-lycorine, and 1,2-*O*-diacetyl-5,6-dehydrolycorine were separated using pH-zone-refinement centrifugal partition chromatography and identified using NMR and GC-MS [77].

#### 5.1.21. *Rhodophiala bifida*

The bulbs of *R. bifida* consisted of montanine, vitattine, hemanth amine, hemanthidine, tazettine, corianicine, and traces of lycorine and O-acetylmontanine [78].

#### 5.1.22. *Zephyranthes species*

Alkloids in *Zephyranthes* species have been reviewed [79]. Alkaloids have been found in *Z. andersoniana*, *Z. candida*, *Z. carinata*, *Z. citrina*, *Z. concolor*, *Z. flava*, *Z. grandiflora*, *Z. lancasteri*, *Z. robusta*, *Z. rosea*, *Z. sulphurea*, *Z. tubispatha*, and *Z. texana*

Eleven new alkaloids identified as six types of 12-acetylplicamine, *N*-deformyl-seco-plicamine, plicamine, 4α-epi-plicamine, seco-plicamine, and lycorine, along with fifteen known alkaloids, were isolated from the whole plants of *Z. carinata* [80]. Also, three new *Amaryllidaceae* alkaloids, named zephycandidines I-III, were isolated from *Z. candida*. Zephycandidines I and III with new framework types represent the first example of 7-phenyl-hexahydroindole and 5,2′-dimethyl-biphenyl-2-ylamine alkaloids, respectively [81].

Alkaloids in seeds [82] and flowers [83] of *Z. flava* were studied. The flowers contain four new alkaloidal phospholipids, 2-*O*-glyarophosphoryllycorine, phosphatidyllycorines, phosphatidylpscudolycorines, and phosphatidyllycorinium methocation, whereas the seeds contain two new alkaloids, zefbetaine and zeflabetaine, together with known alkaloids of crinamine, haemanthamine, lycorine, maritidine, methylpseudolycorine, pretaxettine Haemanthidine, pseudolycorine, narciclassine, pratorimine, kalbreclassine, lycorine-1-*O*-β-D-glucoside, pseudolycorine-1-*O*-β-D-glucoside, betaines, criasbetaine, and ungeremine.

Twenty-six structurally diverse *Amaryllidaceae* alkaloids were isolated from whole plants of *Z. candida*. These include ten novel compounds—zephyranines A to I and 6-*O*-ethylnerinine—as well as two previously undescribed natural products, zephyranthine-6-one and 3-*O*-deacetyl-sternbergine. The study evaluated nine distinct alkaloid frameworks for their acetylcholinesterase inhibitory and anti-inflammatory activities [84].

(+)-Epimaritidine, a new alkaloid from *Z. rosea*, together with known alkaloids crinamine and haemanthamine are reported [85]. Phytochemical investigation of the 95% ethanol extract of the whole plants of *Z. grandiflora* led to the isolation of six new 4α-epi-plicamine-type alkaloids, named zephygranditines A-F, along with three novel 11,12-seco-plicamine-type alkaloids [86].

The bulbs and aerial parts of *Z. concolor* (Lindl.) Benth. & Hook. f., an endemic Mexican species, contain the alkaloids chlidanthine, galanthamine, galanthamine *N*-oxide, lycorine, galwesine, and epinorgalanthamine [87]. Eight alkaloids have been isolated from *Z. citrina* (*Amaryllidaceae*), including oxomaritidine, which is reported for the first time from a natural source [88].

#### 5.1.23. Genus *Hymenocallis*

The genus *Hymenocallis* has 40 species (Table 4), and some species are showcased here. The bulbs of *H. littoralis*, *H. crassifolia*, and *H. caymanensis* contain isolated tazettine and lycorine, whereas *H. occidentalis* contains tazettine, lycorine, and nivaline; and *H. speciosa* has a major component of lycorine with minor components of tazettine, urceoline, hippeastrine, and haemanthamine [34].

In searching the sources of pancratistatin, a promising anticancer agent, *Hymenocallis* species representing a broad geographical selection were investigated. Pancratistatin was isolated from *H. speciosa* (Singapore), *H. variegata* (Singapore), *H. pedalis* (Seychelles), *H. expansa* (Bermuda), and *H. sonoranensis* (Mexico) [89].

From *Hymenocallis x festalis* Hort. Ex Schmarse, a hybrid of *H. longipetala* and *H. narcissiflora*, a new phenanthridine-type alkaloid 3-methoxy-8,9-methylenedioxy-3,4-dihydrophenanthridine (2 mg, from 8 kg of fresh bulbs) and 3-Methoxy-3,4-dihydrotrisphaeridine were isolated [90].

#### 5.1.24. Genus *Ismene*

The genus *Ismene* has six species (Table 4), including *I. amancaes*, *I. hawkesii*, *I. longipetala*, *I. narcissiflora*, *I. vargasii*, and *Ismene x deflexa*. The plant extract of *I. amancaes* contains lycoramine, a galanthamine-type alkaloid, identified by GC-MS [91].

#### 5.1.25. Genus *Lapiedra*

The genus *Lapiedra* has one species (Table 4), *L. martinezii*. From this species, homolycorine N-oxide and O-methyllycorenine N-oxide were isolated, and the authors claimed that they represented the first examples of naturally occurring N-oxides from the *Amaryllidaceae* [92]. From the methanolic extract of *L. martinezii*, a new alkaloid, N-methyl-assoaninium chloride, has been isolated together with hippadine, narcissidine, and ungiminorine [93]. N-Chloromethyl-narcisidinium chloride was also isolated but is probably an artifact formed during the isolation process. In another study on *L. martinezii* by the same group, the known alkaloid ismine, was isolated together with three phenatridine alkaloids, 8,9-methylenedioxophenantridine, N-methyl-8,9-methylenedioxy-6-phenantridone, and N-methyl-8,9-methylenedioxy-phenantridinium chloride [94].

#### 5.1.26. Genus *Leucojum*

The genus *Lapiedra* has two species (Table 4), including *L. aestivum* and *L. vernum*, from which alkaloids have been isolated.

*Leucojun vernum* contains leucovernine, acetylleucovernine, and 9-O-demethylhomolycorine [95].

A new alkaloid, N-(14-methylallyl)norgalanthamine, together with five known alkaloids, *N*-allylnorgalanthamine, galanthamine, epinorgalanthamine, narwedine, and lycorine, were isolated from mother liquors (waste material) obtained after the industrial production of galanthamine hydrobromide from *Leucojum aestivum* leaves [96]. The production of galanthamine from the tissue culture of *Leucojum* leaves was studied [97].

#### 5.1.27. Genus *Lycoris*

The genus *Lycoris* has 25 species (Table 4), and alkaloids were extensively isolated as highlighted below.

The investigation of the 80% EtOH extract of the bulbs of *L. aurea* led to the isolation of six new alkaloids, 2-demethyl-isocorydione, 8-demethyl-dehydrocrebanine, 1-hydroxy-anhydrolycorin-7-one, (+)-1,2-dihydroxy-anhydrolycorine N-oxide, 5,6-dihydro-5-methyl-2-hydroxyphenanthridine, and (+)-8-hydroxy-homolycorine-α-N-oxide, in addition to two known compounds, isocorydione and anhydrolycorin-7-one [98].

Dihydrocaranine, dihydrolycorine, 7-oxodihydrolycorine, 3,4-dihydroanhydrolycorine, norbelladine, 2α-hydroxy-O-methyloduline, 3β-methoxy-6,11-dihydroxycrinane, 3β,11-dihydroxycrinane, 6β-acetoxycrinamine, 3α,6β-diacetyl-bulbispermine, 3α-hydroxy-6β-acetylbulbispermine, 3α-methoxy-6β-acetylbulbispermine, N-demethyl-8α-ethoxypretazettine, and N-demethyl-8β-ethoxypretazettine were also found in *L. radiata* [99].

From the bulbs of *L. radiata*, 2α-methoxy-6-O-ethyloduline, O-demethyllycoramine, and *N*-chloromethyl ungiminorine were isolated as new alkaloids together with the known compounds of radiatine, 2α-hydroxy-6-O-methyloduline, O-ethyllycorenine, O-methyllycorenine, 9-O-demethylhomolycorine, 9-O-demethyl-2α-hydroxyhomolycorine, hippeastrine, lycoramine N-oxide, O-demethyllycoramine, lycoramine, galanthamine N-oxide, sanguine, galanthamine, (−)-epi-zephyranthine, dihydrolycorine, lycorine, hippamine, 4-O-methyllycorine, pseudolycorine, pluviine, pancratinine, (−)-3-*O*-methylpancracine, pancracine, narciclasine, and 2′-deoxythymidine [100].

Four new *Amaryllidaceae* alkaloids—(+)-1-hydroxy-ungeremine, (+)-6β-acetyl-8-hydroxy-9-methoxy-crinamine, (+)-2-hydroxy-8-demethyl-homolycorine-α-N-oxide, and (+)-N-methoxycarbonyl-2-demethyl-isocorydione—along with two known compounds, (+)-6β-acetyl-crinamine and 8-demethyl-homolycorine-α-N-oxide, were isolated from the ethanol extract of the bulbs of *L. radiata* [101]. Also, a new lycorine-type alkaloid, 1-O-(3’*S*)-hydroxybutanoyllycorine, together with four known alkaloids of galanthamine, lycoramine, sternbergine, and ungiminorine were isolated from *L. traubii* [102].

The bulbs of *L. caldwellii* afforded four new alkaloids, (+)-*N*-methoxylcarbonyl-nandigerine, (+)-*N*-methoxycarbonyl-lindcarpine, (+)-10-*O*-methylhernovine *N*-oxide, and (+)-3-hydroxy-anhydrolycorine *N*-oxide [103]. *Lycoris sprengeri* contains lycosprenine, narcissidine, tortuosine, 2α-methoxy-6-*O*-methyllycorenine, lycoramine, montabuphine, and crinasiadine [104].

#### 5.1.28. Genus *Narcissus*

The genus *Narcissus* (commonly known as daffodils) has 97 species (Table 4), and alkaloids have been reported extensively. A total of 92 *Amaryllidaceae* alkaloids were reported up to July 2005 from about 40 wild species, and 100 cultivars of the genus *Narcissus* were isolated and reviewed [105].

The bulbs of *N. bicolor* growing in Spain afforded three new alkaloids, bicolorine, 5,6-dihydrobicolorine, and oxoassoanine-*N*-oxide, whose structures were determined by mass and spectral analyses. Three known alkaloids, pretazettine, 9-*O*-demethylhomolycorine, and 3-epimacronine, were also isolated [106].

The aerial parts of *N. papyraceus* contain three new alkaloids, *O*-methylpapyramine, *O*-methylmaritidine, and 9-*O*-demthylhomolycorine N-oxide, together with lycorine, papyramine, pseudolycorine, homolycorine, and 9-*O*-demethylhomolycorine [107].

The whole plant of *N. radinganorum* contains three *Amaryllidaceae* alkaloids—homolycorine, 8-*O*-demethylhomolycorine, and 9-*O*-demethylmaritidine—with the third compound being reported for the first time [108]. From *N. pallidullus*, mesembrenone and roseine were isolated [109].

Seven alkaloids were isolated from fresh bulbs of *N. angustifolius* subsp. *transcarpathicus*, with nangustine being the 5,11-methanomorphanthridine alkaloid with a C-3/C-4 substitution, and reported for the first time [110]. In another study, eleven alkaloids were isolated from the whole plants of *N. bujei*, with 11-*O*-acetylhaemanthamine and bujeine being reported for the first time [111].

Homolycorine was isolated from *N. confusus* Pugsley, and its chirality was determined by an X-ray crystallographic analysis of the hydrochloride dihydrate [112]. Ismine was isolated from several *Narcissus* species [113]. A study on the isolation and acetylcholinesterase inhibition found four alkaloids—(−)-9-*O*-methylpseudolycorine, (−)-narcissidine, (−)-pancratinine-C, and (+)-9-O-demethyl-2-α-hydroxyhomolycorine—isolated from *N. tazetta* subsp. *tazetta* L. [114].

From the fresh bulbs of *Narcissus* cv. Professor Einstein, a new lycorine-type alkaloid, 7-oxonorpluviine, was isolated together with 23 known alkaloids of masonine, homolycorine, ismine, caranine, galanthamine, narwedine, lycoraminone, pluviine, incartine, galanthine, lycoramine, epinorgalanthamine, norlycoramine, haemanthamine, hippeastrine, epimaritidine, lycorine, tazettine, eugenine, norpluviine, 9-*O*-demethylmaritidine, pancracine, and 9-*O*-demethylhomolycorine [115].

#### 5.1.29. Genus *Nerine*

The genus *Nerine* has nine species (Table 4), including *N. alta*, *N. bowdenii*, *N. humilis*, *N. huttoniae*, *N. laticoma*, *N. masonorum*, *N. platypetala*, *N. sarniensis*, and *N. undulata*.

From 1 kg of *N. undulata*, 0.51 g of lycorine, 0.03 g of ambelline, 0.05 g of undulatine, 0.04 g of base N, 0.07 g of nerispine, and 0.7 g of crispine were obtained [36]. *Nerine bowdenii* Watson contains filifoline, buphanisine, 11-*O*-acetylambelline, ambelline, and undulatine [116].

*N*-Demethylbelladine, 6a-methoxybuphanidrine, and filifoline were isolated and identified as new alkaloids in addition to the known alkaloids of belladine and 6a-methoxybuphanidrine from *N. filifolia* [117]. A new mesembrine-type alkaloid, named sarniensine, was isolated together with tazettine, lycorine, and 3-epimacronine from *N. sarniensis*. Lycorine and 3-epimacronine are reported from this species for the first time [118].

#### 5.1.30. Genus *Pancratium*

The genus *Pancratium* has 14 species (Table 4). This genus has been reviewed in Volume 68 (2010) of *The Alkaloids* [119].

The isolated alkaloids from the genus (the most studied species is *P. mancratium*) were grouped under eight types [119]:

(1) Lycorenine type, which includes hippeastrine, (+)-9-*O*-demethylhomolycorine, 10-norneronine, and pancratinine A;
(2) Lycorine type, which includes pancrassidine, galanthane, hippadine, 3,4-dihydroanhydrolycorine, hihydrocaranine, dihydrolycorine, ungeremine, zefbetaine, ungiminorine N-oxide, and pancratinine D;
(3) Montanine type, which includes pancracine from *P. maritimum* and *P. sickenbergeri*; pancratinine B, and pancratinine C from *P. canariense*;
(4) Narciclasine type, which includes pancratistatin, narciclasine, and its glycosidic derivative;
(5) Tazettine type, which includes tazettine and deoxytazettine from *P. maritimum* and pretazettine from *Pancratium* biflorum;
(6) Galanthamine type, which includes galanthamine, N-norgalanthamine, *N*-formylgalanthamine, habranthine, lycoramine, *N*-norlycoramine, and 3-*O*-acetyllycoramine;
(7) Cranine type, which includes crinine, crinan-3-one, buphanisine, macowine, (−)-3β-methoxy-6,11-dihydroxycrinane, and (−)-3β-11-dihydroxycrinane; and
(8) Haemanthamine, which includes haemanthamine, vittatine, 11-hydroxyvittatine, maritidine, haemanthidine, ent-6-hydroxybufanisine, 8-demethylmaritidine, 9-demethylmaritidine, crinamine, and 6-*O*-methylhaemanthidine.

*Pancratium canariense* possessed 12 alkaloids, including 1-*O*-acetyl-8-norpluviine, ungiminorine, pancratinine, 10-norneronine, littatine, 11-hydroxyvitattine, 6-*O*-methylhaemanthidine, 6-*O*-methylhaemanthidine, and pancracine [109].

Ungeremine and zefbetaine were isolated from Egyptian *P. maritimum* [120]. 11α-hydroxy-*O*-methylleucotamine was isolated for the first time from *P. illyricum* L., which contains eight known alkaloids: lycorine, 2-hydroxyhomolycorine, vittatine, galanthamine, sanguinine, habranthine, leucotamine, and *O*-methylleucotamine [121].

Ungiminorine *N*-oxide was isolated from *P. maritimum*, and the authors claimed that they represented the first examples of naturally occurring N-oxides from the *Amaryllidaceae* [92].

#### 5.1.31. Genus *Phaedranassa*

The genus *Phaedranassa* has eight species (Table 4), including *P. carmiolii*, *P. cinerea*, *P. dubia*, *P. lehmannii*, *P. schizantha*, *P. tunguraguae*, *P. ventricose*, and *P. viridiflora*.

Bulbs (771 g) of *P. dubia* were found to contain a new phaedranamine (12 mg) together with seven known alkaloids: pseudolycorine (28 mg), haemanthamine (15 mg), sanginine (5 mg), epinorgalanthamine (4 mg), galanthamine (5 mg), zefbetaine (8 mg), and ungeremine (6 mg) [122].

#### 5.1.32. Genus *Scadoxus*

The genus *Scadoxus* has four species (Table 4), including *S. puniceus*, *S. cinnabarinus*, *S. membranaceus*, and *S. multiflorus*. Haemanthamine and haemanthidine were isolated from *S. puniceus* [123].

#### 5.1.33. Genus *Sternbergia*

The genus *Sternbergia* has eight species (Table 4), including *S. candida*, *S. clusiana*, *S. colchiciflora*, *S. greuteriana*, *S. lutea*, *S. pulchella*, *S. sicula*, and *S. vernalis*.

Bulbs of *S. clusiani* were found to contain the following seven known alkaloids: lycorine, galanthamine, haemanthamine, haemanthidine, 11-hydroxyvittatine, crinine, and isotazettine [124]. From the bulbs of *S. lutea* a new compound was isolated and named sternbergine, which was elucidated as 1-O-acetylisopseudolycorine. Previously isolated alkaloids lycorine, tazettine, hippeastrine, galanthine, galantham lycorine, galanthamine, hippeastrine, and tazzetine were also found [125].

The crinine-type alkaloids (+)-buphanisine and (−)-siculine were isolated from *S. sicula*, while (−)-epimaritinamine and (−)-mariti namine were found in *S. lutea* [126]. Aerial parts of *S. lutea* isolated 1% lycorine and 0.18% pancratine, as compared to the bulbs of this plant, which has 0.21% lycorine, 0.052% tazettine, and 0.028% pancratine [127]. Twenty-one alkaloids and related compounds were found in *S. colchiciflora*. Ten alkaloids were isolated, and their structures confirmed by NMR, MS, and CD measurements [128]. The *Sternbergia clusiana* of Turkish origin yielded four alkaloids, lycorine, haemant hamine, haemanthidine, and tazettine [129].

#### 5.1.34. Genus *Ungernia*

The genus *Ungernia* has two species (Table 4), including *U. flava* and *U. tadschicorum*.

*Ungernia* alkaloids have been reviewed up to 1980 [130]. About 20 alkaloids representing structural types of lycorine, crinine, lycorenine, galanthamine, and tazettine were isolated from the genus, including *U. tadshicorum*, *U. severzovii*, *U. victoris*, *U. vvedenskyi*, *U. trisphaera*, and *U. ferganica*.

Most of the isolation work was reported by Russian scientists on the *Ungernia* species grown in Russia; for example, ungvedine was isolated from *U. vvedenskyi* [131]. The extracting of 11 kg of powdered dry leaves of *U. spiralis* (moistened with 8% ammonia) with chloroform, treating the extract with 10% sulfuric acid, and alkalizing it with an ammonia solution produced 0.11% lycorine, 0.75 g of galanthamine, 0.3 g of ungeremine, 0.2 g of hippeastrine, and 0.15 g of tazettine [127]. 

#### 5.1.35. Genus *Urceolina*

The genus *Urceolina* has two species (Table 4), *U. microcrater* and *U. peruviana*. The bulbs of *U. miniata* contain 0.13% alkaloids consisting of 52% tazettine, 31% haemanthamine, 4% lycorine, and two new alkaloids in the 4% yield, namely urceoline and urminine [34].

#### 5.1.36. Genus *Worsleya*

The genus *Worsleya* has two species (Table 4), *W. procera* and *W. rayneri*. Fifteen alkaloids from *W. procera* roots were identified by GC-MS, and seven of them were isolated [132]. The seven isolated alkaloids are ismine, trisphaeridine, tazettine, galanthine, lycorine, homolycorine, and albomaculine.

### 5.2. Detected and Identified Amaryllidaceae Alkaloids from the Subfamily Amaryllidoideae

The genera in the subfamily *Amaryllidoideae*, where *Amaryllidaceae* alkaloids are detected or tentatively identified (but not isolated) as summarized in Table 4, are detailed in Table 5 below.

### 5.3. The Genera from the Subfamily Amaryllidoideae with No Amaryllidaceae Alkaloids Reported

Genera in subfamily *Amaryllidoideae*, where *Amaryllidaceae* alkaloids have not been reported (undetected, unidentified, or un-isolated) as summarised in Table 4, are detailed here.

No Amaryllidacea alkaloids were found in the *Amaryllidaceae* alkaloid profiling using GC-MS for the two species *A. cedarbergense* and *A. lanceolatum* in the genus *Apodolirion* (Table 4) and for seven species, *G. afra*, *G. britteniana*, *G. ciliaris*, *G. grandiflora*, *G. lanuginose*, *G. namaquensis*, and *G. verticillate*, in the genus *Gethyllis* (Table 4) [137].

The following genera and species were only found in phylogenetic studies [133]:

(1) Genus Caliphruria (Table 4), three species C. korsakoffi, C. subedentata, and C. teneraz;
(2) Genus Cryptostephanus (Table 4), two species C. haemanthoides and C. vansonii;
(3) Genus Eremocrinum (Table 4), one species E. albomarginatum;
(4) Genus Eucrosia (Table 4), six species E. aurantiaca, E. bicolor, E. dodsonii, E. eucrosioides, E. mirabilis, and E. stricklandii;
(5) Genus Hannonia (Table 4), one species H. hesperidum;
(6) Genus Haylockia (Table 4), one species H. Herb., 1830;
(7) Genus Hessea (Table 4), seven species H. breviflora, H. pilosula, H. pulcherrima, H. speciosa, H. stellaris, H. stenosiphon, and H. zeyheri;
(8) Genus Namaquanula (Table 4), one species N. bruce-bayeri;
(9) Genus Pabellonia (Table 4), one species P. Quezada & Martic;
(10) Genus Plagiolirion (Table 4), one species P. horsmannii;
(11) Genus Rauhia (Table 4), three species R. decora, R. multiflora, and R. staminosa;
(12) Genus Stemmatium (Table 4), one species S. Phil., 1873;
(13) Genus Stenomesson (Table 4), eight species S. aurantiacum, S. chloranthum, S. ecuadorense, S. flavum, S. leucanthum, S. miniatum, S. pearcei, and S. variegatum;
(14) Genus Strumaria (Table 4), ten species S. aestivalis, S. bidentata, S. chaplinii, S. discifera, S. picta, S. salteri, S. spiralis, S. tenella, S. truncate, and S. watermeyeri;
(15) Genus Vagaria (Table 4), two species V. ollivieri and V. parviflora.

## 6. Bioactivities of *Amaryllidaceae* Alkaloids for Drug Discovery

*Amaryllidaceae* alkaloids occur mainly in the subfamily *Amaryllidoideae* and have only recently been reported in another family, *Asparagaceae*, but under the same order of *Asparagales* as the family *Amaryllidaceae*. From *Hosta plantaginea* of the genus *Hosta* in the family *Asparagaceae*, five new benzylphenethylamine alkaloids, hostasine, 8-demethoxyhostasine, 8-demethoxy-10-*O*-methylhostasine, 10-*O*-methylhostasine, and 9-*O*-demethyl-7-*O*-methyllycorenine along with twelve known compounds were isolated [138]. The reason why plants produce alkaloids almost exclusively from the subfamily *Amaryllidoideae* but not *Agapathiodeae* and *Alliodeae* remains unclear, although general evolutionary mechanisms underlying the secondary metabolite diversity are likely at play [139]. It is possible that these alkaloids play a critical role in the plants’ survival, serving ecological or defensive functions. One might even say that nature has generously bestowed these compounds for the benefit of humanity.

The isolation of the alkaloids has been extensively conducted in the genera *Crinum*, *Galanthus*, *Hippeastrum*, *Lycoris*, *Narcissus*, *Pancratium*, and *Zephyranthes*. Lycorine is a very common *Amaryllidaceae* alkaloid, and its effects include the regulation of autophagy; the induction of cancer cell apoptosis; and anti-inflammatory, antifungal, antiviral, antimalarial, and antitumor effects [140]. The clinically used galanthamine has been isolated from the genera *Cyrtanthus*, *Galanthus*, *Leucojum*, *Lycoris*, *Narcissus*, *Ungernia*, *Chlidanthus*, *Crinum*, *Eucharis*, *Eustephia*, *Pancratium*, and *Phaedranassa*. Chromatographic techniques for separation and isolation as well as spectroscopic methods and single-crystal X-ray diffraction techniques for the characterisation of herbal biomolecules [141] are extensively used in the isolation and identification of *Amaryllidaceae* alkaloids. Drug discovery from the *Amaryllidaceae* alkaloids and the family *Amaryllidaceae* plant resources is ongoing and warranted.

The chemistry and bioactivities of *Amaryllidaceae* alkaloids, remarkable natural products gifted by Mother Nature, have garnered significant attention for their potential in drug discovery and therapeutic innovation. These alkaloids, characterized by their structural diversity and complex biosynthesis, are extensively reviewed in the literature for their broad pharmacological relevance [8,142,143]. The discussion that follows is intended as a focused appreciation of their bioactivity, with a particular emphasis on acetylcholinesterase inhibition—a key target in Alzheimer’s disease—as well as their anti-inflammatory, antioxidant, antimicrobial, antidiabetic, and anticancer properties. These multifaceted activities underscore the continued importance of *Amaryllidaceae* alkaloids as promising leads in the search for novel, plant-derived medicinal compounds.

### 6.1. Inhibiting Acetylcholinesterase (Anti-Alzheimer’s)

Acetylcholinesterase (AChE) is an enzyme found in the synaptic cleft (the gap between nerve cells) and neuromuscular junctions. Its main function is to break down the neurotransmitter acetylcholine (ACh) into acetate and choline, terminating the nerve signal. When AChE is inhibited, acetylcholine levels remain elevated, leading to a prolonged stimulation of cholinergic receptors. In the case of Alzheimer’s disease, drugs like galathamine inhibit acetylcholinesterase and boost cholinergic transmission in the brain, improving memory and cognition. Galantamine can stimulate presynaptic and postsynaptic nicotinic receptors, which can then increase the release of neurotransmitters such as ACh and glutamate, directly stimulating the neuronal function [144].

A comprehensive study on *Narcissus tazetta* subsp. *tazetta* L. reported that 11-hydroxygalanthine and narcissidine inhibit acetylcholinesterase significantly [114]. In a related study, 100 micrograms of extracts from *Crinum jagus* (Thomps.), *Crinum × amabile* Donn, *Crinum zeylanicum* (L.) L. (all *Amaryllidaceae*), and *Agapanthus praecox* subsp. *orientalis* (F.M. Leight.) F.M. Leight. (Liliaceae) inhibited the acetylcholinesterase (AChE) activity, as detected using a TLC bioautographic method [145]

Earlier investigations showed that chlidanthine at 2.4 × 10^−5^ M and galanthamine *N*-oxide at 2.6 × 10^−5^ M inhibited electric eel AChE, although both were approximately five times less potent than galanthamine, while galwesine at 10^−3^ M was inactive [87]. Additionally, in testing 23 pure *Amaryllidaceae* alkaloids and 26 extracts from different *Narcissus* species, 7 alkaloids—belonging to the galanthamine and lycorine skeleton types—exhibited AChE inhibitory effects, with sanguinine proving to be the most active and even surpassing galanthamine [146].

In a recent review, the IC_50_ values of alkaloids in major *Amaryllidaceae* alkaloid types inhibiting AChE have been summarized [142]. Anti-Alzheimer’s effects of the alkaloids from *Galanthus* spp. are also reviewed [147]. The AChE inhibitory activity of extracts and compounds from the plants of the *Amaryllidoideae* subfamily has been explored in the literature over the last two decades [148]. The anti-cholinesterase potential of 26 alkaloids from *Urceolina* Rchb., *Clinanthus* Herb., and *Stenomesson* Herb. was also reviewed [149]

### 6.2. Anti-Inflammatory

Lycorine and narciclasine displayed potent effects against pain, swelling, asthma, and arthritis in a recent review summarizing 140 anti-inflammatory principles from *Amaryllidaceae* plants [150]. In a separate study, 51 species from the *Amaryllidaceae* family were identified for their traditional use in treating inflammation across 32 countries [151].

Zephyranine B, haemanthamine, haemanthidine, 11-hydroxyvittatine, and 8-demethoxy-10-O-methylhostasine demonstrated a potent anti-inflammatory activity by inhibiting the LPS-induced nitric oxide (NO) production in RAW264.7 mouse macrophages, with IC_50_ values of 21.3, 4.6, 12.2, 5.6, and 17.4 μM, respectively; a structure–activity relationship (SAR) analysis and molecular docking studies indicated that an effective acetylcholinesterase (AChE) inhibition requires interactions with key active site residues, Trp286 and Tyr337 [84].

Galanthamine, lycorine, narciclasine (lycoricidinol), and crinamine have been shown to act as anti-inflammatory agents, targeting the cholinergic anti-inflammatory pathway [12]. Narciclasine inhibits leukocyte–endothelial interactions by blocking endothelial activation processes through the loss of TNF receptor 1 [152].

Additionally, five *Amaryllidaceae* alkaloids—4,8-dimethoxy-cripowellin, 9-methoxy-cripowellin, 4-methoxy-8-hydroxy-cripowellin, and cripowellin—exhibited a significant inhibition of COX-1 (>64%) and COX-2 (>90%), which was comparable to positive controls [48]. Narciclasine has also been demonstrated to exert profound anti-inflammatory effects in vivo [153]. Norbelladine at 0.25 μM inhibited the COX-1 and COX-2 activity by 51% and 25%, respectively, and at 10 μM inhibited the NF-κB activation by 23% [154].

### 6.3. Antioxidant

9-methoxy-cripowellin and 4-methoxy-8-hydroxy-cripowellin exhibited a notable antioxidant activity in the ABTS^.+^ and DPPH tests [48]. Norbelladine, at a concentration of 10 μM, was able to quench the DPPH-radical by 31% and reduce superoxide radicals generated from xanthine oxidase by 33% [154]. Additionally, lycorine administered intraperitoneally at doses of 1.0 mg/kg and 1.5 mg/kg significantly reduced rat paw oedema induced by carrageenan, achieving a 53.45% and 36.42% inhibition, respectively, compared to the 95.70% inhibition observed with indomethacin (3 mg/kg, i.p.) [155]. Antioxidant activities of montanine [156], five alkaloids from *Crinum* latifolium [48], and the methanolic extracts of *Hieronymiella peruviana* bulbs [71] were reported.

### 6.4. Antimicrobial

The methanol extract of *Crinum jagus* demonstrated a broad-spectrum antimicrobial activity, with minimum inhibitory concentration (MIC) values ranging from 1 to 500 mg/L against a panel of microorganisms. Notably, the extract showed a strong activity against *Mycobacterium smegmatis*, *Salmonella typhi*, and *Pseudomonas aeruginosa*, which are typically difficult to eradicate. The extracts were administered orally to rats at doses of 30, 150, and 300 mg/kg [157]. Similarly, 9-methoxy-cripowellin and 4-methoxy-8-hydroxy-cripowellin exhibited a significant antimicrobial activity, displaying IC_50_ values below 0.50 mM against eight tested bacterial strains [48]. Antimicrobial activities of montanine were reported [156].

### 6.5. Antidiabetic

Galantamine exhibits antidiabetic effects by stimulating the cholinergic pathway through acetylcholinesterase (AChE) inhibition and activating the efferent vagus nerve via its action as α7 nAChR agonist. Acting as a neural bridge between the liver, pancreatic cells, and adipose tissue, galantamine influences the insulin secretion, pancreatic cell mass, energy expenditure, glucose metabolism, hepatic glucose and glycogen synthesis, systemic insulin sensitivity, and fat distribution between the liver and peripheral tissues [158].

### 6.6. Anticancer

Five *Amaryllidaceae* alkaloids:4,8-dimethoxy-cripowellin, 9-methoxy-cripowellin, 4-methoxy-8-hydroxy-cripowellin, and cripowellin—demonstrated a potent cytotoxicity against seven lung cancer cell lines with IC_50_ values below 30 nM [48]. Similarly, four alkaloids—(+)-1-hydroxy-ungeremine, (+)-6β-acetyl-8-hydroxy-9-methoxy-crinamine, (+)-2-hydroxy-8-demethyl-homolycorine-α-N-oxide, and (+)-N-methoxylcarbonyl-2-demethyl-isocorydione—exhibited significant cytotoxic activities against eight tumour cell lines, including CCF-STTG1, CHG-5, SHG-44, U251, BGC-823, HepG2, and SK-OV-3 [101].

In another study, crinine, 6-hydroxybuphanidrine, and 6-ethoxybuphanidrine showed antiproliferative effects against human tumour cell lines, with crinine being the most potent (IC_50_ = 14.04 μM against HL-60/Dox). Crinine also induced apoptosis in a dose-dependent manner in HL-60 and MDA-MB-231 cell lines [159]. Using the noncancerous cell line MRC-5 human fibroblasts as a control and the WST-1 metabolic activity assay, the growth of nine cancer cell lines was inhibited by pancracine (a montanine-type alkaloid), with IC_50_ values ranging from 2.20 to 5.15 µM [115].

In a recent review, the IC_50_ values of major *Amaryllidaceae* alkaloid types inhibiting the growth of various cancer cell lines have been summarized [142]. Cytotoxic aspects of *Amaryllidaceae* alkaloids in *Galanthus* species have been recently reviewed, including the structural types of galanthamine, homolycorine, haemanthamine, galanthindole, graciline, and other types [61].

### 6.7. Other Bioactivities

*Amaryllidaceae* alkaloids have demonstrated a wide range of bioactivities. Several studies have reported antiviral, antiparasitic, and DNA-binding activities. Lycorine inhibited the Poliomyelitis virus at concentrations as low as 1 mg/mL, although higher concentrations (>25 mg/mL) were cytotoxic [43]. A broader review of the antiviral effects of *Amaryllidaceae* plants [160] and *Amaryllidaceae* alkaloids [161] was also conducted recently.

The alkaloids ungeremine, pseudolycorine, and haemanthamine exhibited a good antiparasitic activity against *Trypanosoma brucei rhodesiense*, *T. cruzi*, and *Plasmodium falciparum*, with IC_50_ values below 3.66 μM [122]. Additionally, lycorine and 1,2-di-O-acetyllycorine, isolated from *Brunsvigia littoralis*, showed a significant antimalarial activity against *Plasmodium falciparum*, while crinamine, with an α-configuration ethano bridge, exhibited potent antimalarial properties in QSAR studies [162]. Furthermore, the DNA binding activity of twenty *Amaryllidaceae* alkaloids across various skeletal types revealed that nine alkaloids showed >90% binding, comparable to vinblastine, and three additional compounds demonstrated moderate activity [163].

Meanwhile, other pharmacological activities have been identified for *Amaryllidaceae* alkaloids. Lycorine caused a significant increase in the contractility and heart rate in isolated perfused guinea pig hearts, an effect abolished by propranolol, suggesting the β-adrenergic receptor stimulation [124]. Lycorine also exhibited hepatoprotective effects at a dose of 2.0 mg/kg i.p. against carbon tetrachloride-induced acute liver toxicity in rats [155].

## 7. Conclusions

This review has surveyed the occurrence and bioactivities of *Amaryllidaceae* alkaloids across 62 genera plus 24 subgenera from the three subfamilies—*Agapanthoideae*, *Allioideae*, and *Amaryllidoideae*—within the family *Amaryllidaceae*. Species were identified based on listings from the National Center for Biotechnology Information (NCBI) Taxonomy Browser, with the literature of the occurrence and bioactivities sourced through PubMed and the Chemical Abstracts Service (CAS) SciFinder-n platform. More than 600 *Amaryllidaceae* alkaloids have been isolated, predominantly from 36 of the 58 genera in the *Amaryllidoideae* subfamily. These alkaloids exhibit a wide range of biological activities, including acetylcholinesterase inhibition and anti-inflammatory, antioxidant, antimicrobial, antidiabetic, and anticancer effects. Given the rich chemical diversity and bioactivity of *Amaryllidaceae* alkaloids and many unexplored genera and species, the further study of *Amaryllidaceae* species and alkaloids is warranted to support the discovery and development of novel therapeutic agents.

## Figures and Tables

**Table 1:** The family *Amaryllidaceae* with three subfamilies and over one thousand species.

Subfamily	Genus	Species ^a^
*Agapanthoideae*	*Agapanthus*	5 + unclassified
*Allioideae*	*Allieae*	524 + unclassified
	*Gilliesieae* (+9 subgenera ^b^)	75 + unclassified
	*Tulbaghieae* (+2 subgenera ^c^)	18 + unclassified
*Amaryllidoideae*	58 genera (+13 subgenera ^d^)	592 + unclassified
Total	62 genera (+24 subgenera)	1214 + unclassified

^a^ Based on “Taxonomy ID 4668, *Amaryllidaceae*” by the National Center for Biotechnology Information (website accessed to 31 January 2025). The variation in the species is not counted. ^b^ See Table 3 for the names of 9 subgenera. ^c^ See Table 3 for the names of 2 subgenera. ^d^ See Table 4 for the names of 13 subgenera.

**Table 2:** The subfamily *Agapanthoideae* and *Amaryllidaceae* alkaloids.

Genera	Species ^a^	*Amaryllidaceae* Alkaloids ^b^
*Agapanthus*	*A. africanus* *A. campanulatus* *A. caulescens* *A. coddii* *A. praecox*	In all five species, no *Amaryllidaceae* alkaloids were reported. *A. campanulatus* (root extract) showed positive alkaloid tests [13].

^a^ Based on “Taxonomy ID 4668, *Amaryllidaceae*” by the National Center for Biotechnology Information (website accessed to 31 January 2025). ^b^ Based on the key word search in SciFinder^n^ using species names + *Amaryllidaceae* and/or alkaloids.

**Table 3:** The subfamily *Allioideae* and *Amaryllidacea* alkaloids.

Genera	Species (Numbers) ^a^	*Amaryllidaceae* Alkaloids ^b^
*Allieae*	*Allium* spp. (524)	No *Amaryllidaceae* alkaloids were found. Other alkaloids were reported [14,15,16].
*Gilliesieae*	Under 9 subgenera: *Beauverdia* spp. (4) *Gilliesia* spp. (7) *Latace* spp. (1) *Leucocoryne* spp. (19) *Miersia* spp. (5) *Nothoscordum* spp. (20) *Speea* spp. (2) *Tristagma* spp. (15) *Zoellnerallium* spp. (2)	No *Amaryllidaceae* alkaloids were reported among a total of 75 species.
*Tulbaghieae*	Under 2 subgenera: *Prototulbaghia* spp. (1) *Tulbaghia* spp. (17)	Among a total of 18 species *T. cernua* and *T. ludwigiana* extracts (1 mg/mL ethanol) inhibited AChE [17].

^a^ Based on “Taxonomy ID 4668, *Amaryllidaceae*” by the National Center for Biotechnology Information (website accessed 31 January 2025). ^b^ Based on the key word search in SciFinder^n^ using species names + alkaloids or *Amaryllidaceae* alkaloids.

**Table 4:** Subfamily *Amaryllidoideae*: genera and species with isolated, detected, or no reported *Amaryllidaceae* alkaloids.

Genus	Spp. ^a^	AA ^b^	Genus	Spp. ^a^	AA ^b^
*Acis*	8	D ^c^	*Hymenocallis*	40	Iso
*Amaryllis*	3	Iso ^d^	*Ismene*	6	Iso
*Ammocharis*	6	Iso	*Lapiedra*	1	Iso
*Apodolirion*	2	- ^e^	*Leptochiton*	1	-
*Boophone*	3	Iso	*Leucojum*	2	Iso
*Brunsvigia*	8	Iso	*Lycoris*	25	Iso
*Caliphruria*	3	-	*Namaquanula*	1	-
*Calostemma*	3	Iso	*Narcissus*	97	Iso
*Carpolyza*	0	-	*Nerine*	9	Iso
*Cearanthes*	0	-	*Pabellonia*	0	-
*Chlidanthus*	2	D	*Pancratium*	14	Iso
*Clinantheae:* ^f^			*Phaedranassa*	8	Iso
*Clinanthus*	6	Iso	*Plagiolirion*	1	-
*Pamianthe*	2	-	*Proiphys*	2	D
*Paramongaia*	4	-	*Pyrolirion*	3	D
*Clivia*	9	Iso	*Rauhia*	3	-
*Crinum*	68	Iso	*Scadoxus*	4	Iso
*Crossyne*	2	Iso	*Shoubiaonia*	1	-
*Cryptostephanus*	2	-	*Stemmatium*	u	-
*Cybistetes*	0	-	*Stenomesson*	8	-
*Cyrtanthus*	44	Iso	*Sternbergia*	8	Iso
*Eremocrinum*	1	-	*Strumaria*	10	-
*Eucharis*	9	Iso	*Ungernia*	2	Iso
*Eucrosia*	6	-	*Urceolina*	2	Iso
*Eurycles*	1	Iso	*Vagaria*	2	-
*Eustephia*	1	Iso	*Worsleya*	2	Iso
*Galanthus*	25	Iso			
*Gethyllis*	7	-			
*Griffinia*	7	D			
*Haemanthus*	12	Iso			
*Hannonia*	1	-			
*Haylockia*	1	-			
*Hessea*	7	-			
*Hieronymiella*	2	Iso			
*Hippeastreae:* ^g^					
*Famatina*	0	-			
*Hippeastrum*	29	Iso			
*Paposoa*	1	-			
*Phycella*	16	D			
*Placea*	0	-			
*Rhodolirium*	3	Iso			
*Rhodophiala*	1	Iso			
*Sprekelia*	1	Iso			
*Traubia*	1	0			
*Zephyranthes*	36	Iso			

Notes: ^a^ Spp., means species numbers in the corresponding genera or subgenera based on “Taxonomy ID 4668, *Amaryllidaceae*” by the National Center for Biotechnology Information (website accessed to 31 January 2025). ^b^ AA, means *Amaryllidaceae* alkaloids surveyed using a combination of key words searched in SciFinder^n^ using species names + alkaloids or “*Amaryllidaceae* alkaloids” in the CAS SciFinder^n^. ^c^ D, means AA detected or identified but not isolated. ^d^ Iso, means AA was isolated. ^e^ -, means AA not reported (not detected, not identified, and not isolated). ^f^ Genus *Clinanthea* contains three subgenera: *Clinanthus*, *Pamianthe*, and *Paramongaia*. ^g^ Genus *Hippeastreae* contains 10 subgenera: *Famatina*, *Hippeastrum*, *Paposoa*, *Phycella*, *Placea*, *Rhodolirium*, *Rhodophiala*, *Sprekelia*, *Traubia*, and *Zephyranthes*.

**Table 5:** Subfamily *Amaryllidoideae*: genera and species with detected or tentatively identified (but not isolated) *Amaryllidaceae* alkaloids.

Genus		Species		Detection or Identification		Reference
*Acis*		*A. autumnalis*; *A. fabrei*; *A. longifolia*; *A. nicaeensis*; *A. rosea*; *A. tingitana*; *A. trichophylla*; *A. valentine*		*Acis valentina* NR349 contained glanathamine-type alkaloids, and *A. autumnalis* NR346 contained lycorine-type and other *Amaryllidaceae* alkaloids.		[133]
*Griffinia*		*G. alba*; *G. espiritensis*; *G. gardneriana*; *G. hyacinthine*; *G. nocturna*; *G. parviflora*; *G. rochae*		*Griffinia nocturna* was studied by UPLC-ESI-MS) for simultaneous analysis of galantamine, pseudolycorine, sanguinine, and narciclasine.		[134]
*Phycella (subgenerus of Hippeastreae)*		*P. angustifolia: P. arzae*; *P. australis*; *P. chilensis*; *P. cyrtanthoides*; *P. aff. cyrtanthoides Garcia 4163*; *P. davidii*; *P. germainii*; *P. herbertiana*; *P. ignea*; *P. lutea*; *P. maulensis*; *P. ornate*; *P. aff. ornata Garcia 726*; *P. scarlatina*		Chloroform basic extracts from *P. herbertiana* contained galanthamine detected by GC-MS.		[135]
*Proiphys*		*P. amboinensis*; *P. cunninghamii*		In *P. amboinensis* ext., haemanthamine and lycorine were identified and trace amounts of alkaloids of the lycorine and homolycorine types were found.		[35]
*Pyrolirion*		*P. albicans*; *P. cutleri*; *P. tubiflorum*		Leaves of *P. albicans* analysed by GC-MS were found to contain galanthamine, chlidanthine, tazettine, and lycorine, and those in the bulbs were galanthamine, *N*-demethylgalanthamine, vittatine/crinine, montanine, pancracine, sternbergine, lycorine, and hippeastrine.		[136]
